# Highlights of the twelfth annual meeting of the National Cancer Research Institute (NCRI), 6–9 November 2016, Liverpool, UK

**DOI:** 10.3332/ecancer.2017.719

**Published:** 2017-02-13

**Authors:** Linda Cairns, Audrey Nailor, Lisa Whittaker

**Affiliations:** 1 European Institute of Oncology, Via Giuseppe Ripamonti, 435, 20141 Milano, Italy; 2 Cancer Intelligence, 154 Cheltenham Road, Bristol, B56 5RL,UK; 3 Research Engagement Officer, Tenovus Cancer Care, Gleider House, Ty-Glas Rd, Cardiff CF14 5BD, Wales, UK

**Keywords:** psychosocial care, cancer prevention, cancer stem cells, obesity

## Abstract

The annual meeting of the National Cancer Research Institute (NCRI), held in Liverpool, UK, is a multidisciplinary conference. The meeting generally outlines research trends for the coming year and is aimed at cancer professionals at every level. The most important themes discussed for this conference was that of cancer stem cells. Alongside once again prominence was given to topics of cancer evolution and the role of social prevention programmes like previous years.

## Introduction

As the Chair, Professor Caroline Dive of the Cancer Research UK Manchester Institute, UK puts it, the programme spans the breadth of cancer research, from basic science to public health.The NCRI meeting is noted for bringing together the diverse cancer stakeholders of the United Kingdom under one roof.

Our correspondents have reported on a selection of the sessions and themes from 2016, especially those which were chosen as particular highlights of the conference.

## Stem cells and cancer

The genetic programmes that control development, renewal, and regeneration of the lung have been the interest of Mark Krasnow of the Stanford University School of Medicine, California, USA for many years. At the plenary lecture, Dr Krasnow elucidated the mechanisms that control the progression of lung progenitors along distinct lineages into mature alveolar cell types.By separating epithelial lung cells into single-cell resolution, he has been able to reconstruct the dynamic gene expression pattern of the different cell types of the lung. The alveolar progenitor cell gives rise to type 1 or 2 cell fates. This is dependent on FGF signalling.

By expressing the Cre recombinase in the progenitor cells of mice, they were able direct the development of new type 2 alveoli. This single-cell approach can be used to powerfully delineate molecularly distinct cell types, characterise progenitors and lineage hierarchies, and identify lineage-specific regulatory factors.

Sheila Singh of McMaster University, Ontario, Canada presented on cancer stem cells and the therapeutic targeting of childhood medulloblastoma. Clinical trials for recurrent medulloblastoma (MB) patients who no longer respond to therapy are based on genomic profiles of the primary tumour, although MBs are highly genetically divergent from their primary tumour.

Thus, it is likely that treatment targets tumours that have already evolved and may not respond to the treatment. Although the level of differentiation is a good indicator of prognosis, there are also underlying molecular subtypes associated with signalling pathways such as Wnt.

Dr Singh’s lab has been studying the signalling pathways in the development of the tumour, such as the epigenetic regulator BM1, and the strategic Wnt activation of BMI1. They found that BMI1 expression is a poor prognostic marker. To study this, her group took cells from the human tumour and injects them into mouse models creating human MB xenografts.

These xenograft models capture the clonal evolution of MB cell populations in response to therapy thereby allowing for comprehensive and serial profiling of tumour clones by regular time point sampling of tumours throughout the course of our mouse-adapted therapy. By reducing BMI1, with a BMI1 inhibitor, we can kill tumour cells in these models.

Future clinical oncology trials will most likely begin with relapsed patients, therapeutic targets from comparative analyses in primary and matched-recurrent tumours offer the greatest clinical yield and hopefully allow better therapeutic choice.

Dominique Bonnet's, of the Francis Crick Institute, London, UK, presentation was on the interactions of leukaemic stem cells with their microenvironment. Acute myeloid leukaemia (AML) is characterised by the unrestrained proliferation of blast cells resulting in deregulation of normal haematopoiesis. Leukaemic stem cells (LSC) depend upon both cell-intrinsic and extrinsic regulatory signals generated by their surrounding microenvironment for their survival and proliferation.

Recent experimental findings in diverse model systems have challenged this view, implicating different stromal cells of the bone marrow in disease pathogenesis. Thus, it is now accepted that leukaemic haematopoiesis can turn the BM niche into a 'leukaemic niche'which promotes LSC function and impairs the maintenance of normal HSC.

However, much remains to be understood about how different leukaemic cells impacts the BM microenvironment, and in turn how changes in the activity of specific BM niche cells contribute to AML pathogenesis. Alterations in the dialogue between leukaemic and stromal cells are involved in leukemogenesis and various models have confirmed this. Dr Bonnet has shown that novel interactions between AML cells and mesenchymal stroma can influence tumourigenic processes such as glucose metabolism and upregulation of certain genes.

In a model to dissect human stroma and AML interaction *in vivo*, her group found that if they knock down MCT-1in the MSC that this impedes AML engraftment. She could rescue MCT4-knock down effect on AML by co-injected SH-control AML.

She concluded her excellent lecture by stating that AML is able to remodel the MSC niche through HIF1a activation via lactate secretion. She stated that targeting MCT4 in AML or MCT1 in MSC blocked AML-MSC communication and abrogated leukaemoenesis. It increased lactate in the AML bone marrow, inhibited normal haematopoiesis via blocking HSC in G0, and that lactate inhibition rescued normal haematopoiesis in AML engrafted mice.

## The 'nanny state' session—prevention at the population level

Over 40% of cancer cases in the UK are linked to preventable risk factors such as smoking, diet, and alcohol use.

Ann McNeill of King's College London, London, UK presented an excellent talk on standardised plain packaging for tobacco products. Tobacco is the leading cause of preventable cancers worldwide, and tobacco advertising, including packaging, directly contributes to increased cancer rates. Plain packaging and standardised packaging reduce the attractiveness of tobacco products. Packaging in the UK, for example, is not only plain but contains distressing imagery and warning messages. Dr McNeill noted that countries hoping to introduce standardised packaging face specific challenges as essentially there is no way to perform randomised controlled trials on the effects of plain packaging. Thus, it is difficult to make evidence-based recommendations about the reduction in cancer rates that nations can expect after introducing standardised tobacco packaging. Further, tobacco industries and linked policymakers are strongly opposed to the introduction of plain packaging.

Tim Stockwell of the University of Vancouver, British Columbia, Canada laughingly referred to the session as the 'nanny state' session. He presented on minimum unit pricing for alcohol, a controversial strategy that he says may be 'the most cost-effective cancer prevention strategy of all.' Alaska, which has implemented this practice, has experienced a predictable drop in alcohol-related cancers.

The carcinogenic element of alcohol, ethanol, is usually clearly indicated on the packaging of alcohol as buyers wish to know how many 'alcohol units' are in each serving. Minimum unit pricing involves charging a tax per unit of alcohol such that low-ethanol alcohol is cheaper to buy. Dr Stockwell argued that this is the most effective measure to prevent alcohol-related cancers. Minimum unit pricing is fairly acceptable to the public, is readily understandable, and is considered better than a flat tax or universal price increases. Perhaps surprisingly, this being the reason that this intervention is supported by some areas of the alcohol industry.

'Canada has very little awareness of alcohol being a cancer-causing product, and I think this is also true in the UK', he said.

Susan Jebb of the University of Oxford, Oxford, UK described policies to treat and prevent obesity. 'Prevention of obesity has been a poor relation when it comes to cancer', she said. There are socioeconomic factors at play; people of higher socioeconomic status are less likely to be obese and more likely to engage in physical exercise for fun, resulting in stratification of cancer risk.

Because food is cheap and accessible—and ought to remain so—methods such as tax increases on food are not a viable way to prevent obesity-related cancers. Interventions that have some success include reformulation or making foods healthier. Residents of the UK have likely not noticed the salt reduction in cornflakes in recent years—and the salt content of bread has dropped by 50% or more depending on type and brand. Reformulation initiatives focused on sugar will be another key to harm reduction. Taxes and levies on non-essential products, such as soft drinks, may also be effective.

Brief weight-loss interventions including those of fad diets are not effective, whereas weight loss programmes are. These should be incorporated into a comprehensive nationwide strategy.

## Early detection and prevention

The cost of treating cancer that is diagnosed early is much less than later treated cancers, and more importantly the prognosis is much better. This session covered some of the implications for early detection and prevention.

Dennis Yuk-Ming Lo of the Chinese University of Hong Kong, Hong Kong, China presented on the use of plasma DNA as a diagnostic tool. Dr Lo has been interested in the diagnostic applications of plasma DNA since the late 1990s. His group developed non-invasive prenatal testing (NIPT) which has been used by millions of pregnant women. The fetal DNA is released into the mother’s blood stream but is quickly cleared after birth. One way to distinguish the fetal DNA from the mother’s DNA is by size; the fetal DNA is shorter than the mother’s because it lacks a linker region.

The success of this NIPT has led to attention focused on the use of plasma DNA in cancer detection. Dr Lo is particularly interested in early screening for liver cancer (HCC), but they are trying to generalise plasma DNA-based cancer screening to other cancer types too through the use of genomewide sequencing of plasma DNA. His group has found that the tumour DNA is also shorter than that of normal DNA. They found it in one case of a pregnant woman with cancer when the cancer was detected incidentally using NIPT.

This plasma DNA comes from dying cells, and hence from these liquid biopsies we can start to screen for cancer. 'Our group has particular interest in the early screening of nasopharyngeal carcinoma (NPC)', Dr Lo said. He also said 'We have developed a method based on the measurement of plasma Epstein-Barr virus (EBV) DNA'.

The group has found that if a patient is operated upon for NPC, the levels of plasma DNA drop in 'just 90 minutes'.

A large study using plasma EBV DNA to screen for NPC is underway and should be completed this year.

'Our next goal is to attempt to generalise plasma DNA-based cancer screening to other cancer types through the use of genomewide sequencing of plasma DNA', Dr Lo said. He further said 'Plasma DNA is a treasure trove for molecular diagnostics'.

Harry de Koning of the University of Glasgow, Glasgow, UK presented an update on Prostate-Specific Antigen (PSA) screening which is one of the best-known early detection screening methods. The European Randomised Study of Screening for Prostate Cancer (ERSPC) showed that PSA-based screening results in a 27% prostate cancer mortality reduction.

Although there are concerns on the harms of over-diagnosis and overtreatment, Dr de Koning argues that it has been shown that the benefits can outweigh the harms. 'Weighing up the benefits against harms in 1000 men, there are 56 life years gained', he said, explaining that with a cost-effectiveness study in a cohort of men aged 55–59, including active surveillance for low-risk men, is increases life years gained. However, he noted, it is important not to screen men who are older i.e., PSA screening should stop at around 65 years of age. This was a key take-home message. He also noted that men should be informed of the unfavourable side effects of prostate cancer screening.

In the future, further improvements are expected for active surveillance and in screening discrimination between indolent and significant disease because of new biomarkers and magnetic resonance imaging.

Rebecca Fitzgerald of the MRC Cancer Cell Unit, University of Cambridge, Cambridge, UK presented on early diagnosis of oesophageal cancer. Cancer of the oesophagus is a global problem with high mortality because of late diagnosis. If dysplasia is found then it can now be easily treated with radiofrequency ablation. Dr Fitzgerald explained how the use of the Cytosponge, in conjunction with biomarkers, will lead to precision for early diagnosis of this disease.

The accessibility of the oesophagus makes it possible to collect cells non-endoscopically using the Cytosponge and apply biomarkers on the retrieved material to detect pre-cancerous lesions. This sponge will have cells from the Barrett’s and also the healthy oesophagus.

The progress made using a variety of genetic and protein biomarkers to diagnose metaplasia and assess the degree of dysplasia in Barrett’s and squamous cell dysplasia and which patients need to be followed is remarkable. For e.g. P53, a prevalent mutation in oesophageal cancer, is a good distinguisher between benign and invasive disease.

The test needs improvements, but this lecture gave hopeful prospect, i.e. better outcomes can be expected for a cancer that was traditionally diagnosed at a late stage.

## Artificial cells as delivery mechanisms for targeted therapy

Oscar Ces of Imperial College London, London, UK presented a workshop on artificial cells (protocells) which have the potential to deliver 'smart' cancer therapy.These protocells consist of water droplets surrounded by lipids in a compartmentalised system which are stable for long periods and are good for drug efficacy studies. These protocells can have numerous compartments. They can be used for ‘cascade reactions’ that would allow drugs to be produced at the site of action, i.e. at the tumour. Ces’s research group are now taking real cells and putting them inside the protocell. The real cell performs reactions and the products (treatments) are released.

A development challenge for the protocell is quality control. For example, there are some variations in cell response to molecular therapeutics. So by using single protocell, Ces hopes to study how individual drugs work at the single cell level.

## Adaptive responses to therapy

Dr Lieping Chen of Yale University, Connecticut, USA presented a plenary lecture on adaptive resistance and anti PD-1/PD-L1 therapy.

Broadly immune responses are controlled by immunomodulatory pathways which can positively or negatively influence immune responses. Defective immunity in the tumour microenvironment impairs an immune response against the tumour. Cancer tissues develop local mechanisms that impair entry or function of immune cells. Dr Chen’s group started to look for immunomodulators in the tumour microenvironment and among other molecules they identified PDL-1. The PD-1/PD-L1 (B7-H1) immunomodulatory pathway can modulate immune responses. The selective expression of PD-L1/B7-H1 in tumour microenvironment and subsequent interaction with PD-1 on T cells is demonstrated to be a major mechanism of losing T-cell immunity in tumour sites in a significant fraction of cancer patients. So Dr Chen’s group started to develop an anti-PD therapy.

Monoclonal antibodies which block this pathway can induce tumour regression. The group found that anti-PD therapy caused regression of melanoma and other solid tumours with a durable response. The treatment is well-tolerated with acceptable toxicity. At least 15 tumour types are in clinical trials for anti-PD therapy. Three drugs have been approved to date including pembrolizumab. A better understanding of the adaptive resistance mechanism underlying response and resistance in anti-PD-1/PD-L1 therapy will be essential for better cancer therapy, and in particular, by following the three principles:

- Targeting the tumour microenvironment
- Repairing tumour-induced immune defects
- Resetting positive tumour immunity

Dr Chen stated that in the future this therapy may be used as first line therapy especially if researchers can identify a biomarker. It may be used in combination therapy. However, we need to better understand and overcome cancer resistance before this can improve patient outcomes.

## Precision oncology and complexity

'Embrace complexity'! was the take-home message of the plenary lecture presented by Rob Bristow of the Ontario Cancer Institute, Ontario, Canada. Prostate cancer is a heterogeneous disease with failure rates approaching 30–40% after precision radiotherapy or surgery, and Dr Bristow’s group is trying to understand this heterogeneity. Relapses in prostate cancer are probably because of pre-existing occult metastases at the time of local treatment. He stated 'It would be good to be able to identify these patients and direct them for more intense therapy'.

Genomic analyses have demonstrated that heterogeneity in mutations, copy number alterations,and methylation amongst lesions that have the same Gleason Score will influence the aggression of these tumours. Using a whole genome sequencing (WGS) approach, Dr Bristow’s group have split men into different genetic sub-groups using these features that are independent prognostic variables for metastatic disease. They developed a bespoke signature at the DNA level to predict patient prognosis, again based on copy number aberrationsand other features such as methylation. Dr Bristow calls this holistic approach 'multimodal genetics.'

The precision can be refined by adding in information regarding the tumour microenvironment, e.g. hypoxia. All of these biomarkers can be used to decide on the aggressiveness and type of therapies offered to patients with potential occult metastases. Dr Bristow believes that this multimodal complex system will improve precision oncology in prostate cancer.

## Cancer genome instability session

Paul Workman of the Institute for Cancer Research, London, UK presented on drug discovery and development to overcome cancer evolution and drug resistance.Traditional chemotherapy is slowly being taken over by innovative precision drugs that counteract effects of particular cancer genes that drive individual cancer –i.e., targeted therapies.

Priorities for drug development include extending the druggable cancer genome to ensure we have agents for all key pathways and models; discovering and using the most effective drug combination drugs; outsmarting adaptation and feedback loops; and devising strategies to combat evolution, heterogeneity, and drug resistance.

A number of the targeted drugs in use were discovered at the Institute for Cancer Research and progressed to the clinic, including abiraterone, and inhibitors of oncogenic protein kinases and PI3 kinase.

'Only 5% of cancer genes are actually druggable, so 95% do not have chemical inhibitors active on them', Dr Workman said. 'This needs to become a priority'.

He gave the examples such as the HSP90 inhibitor luminespib which is showing activity in various cancers, HSF1 targeting as a powerful modifier of carcinogenesis (potential in ovarian cancer; and the APOBEC.

There are many ways by which resistance develops to targeted drugs. These include biochemical rewiring, genetic instability, Darwinian selection, and cellular heterogeneity. 'Overcoming drug resistance is the greatest challenge that we now face in the cancer clinic', Dr Workman concluded.

Nnenna Kanu of University College London, London, UK further explored the potential of APOBEC in her lecture on APOBEC3 mutagenesis in breast cancer. The APOBEC3 family of cytidine deaminases mutate the cancer genome in a range of cancer types. 'We have studied the molecular basis for APOBEC3 expression and activation in HER2 breast cancer', she said. She also said, 'We found that HER2 amplification correlates with APOBEC3 mutagenesis in cell lines'. The silencing of HER2 caused APOBEC expression to be reduced.

To understand better what HER2 is doing, her group studied HER2-enriched breast carcinomas which were found to display evidence of increased levels of replication stress associated DNA damage *in vivo*. When the team induced replication stress through drug exposure, APOBEC3B expression increased.

APOBEC3 activation can be attenuated through repression of oncogenic signalling through exogenous nucleoside supplementation.

Dr Kanu proposed a link between the oncogene, the loss of tumour suppressor gene, and replication stress with APOBEC3 activity. This may explain how cytidine deaminase-induced mutagenesis might be activated in tumourigenesis and limited therapeutically.

Serena Nik-Zainal of the Wellcome Trust Sanger Institute, London, UK presented on mutational signatures in cancer evolution. Mutational signatures are the result of our biological processes and our lifestyle—for example, smoking produces mutational signatures.

Focusing on breast cancer, both ER+ and ER- from a cohort of 560 whole genome sequenced breast cancers, Dr Nik-Zainal’s group found that five mutational signatures were very common and another severe were less rare. These signatures are associated with various mutations;'quite a lot' were related to age and also APOBEC.

'No two patients have the same signature', she said.

By doing whole genome profiling of individual patients, researchers can gain information regarding mutational status, genomic instability, and response. Mutational signatures can explain the subclonal architecture of the cancer genome, distinguishing clinically relevant subpopulations in breast cancer.

## Meeting the psychosocial needs of people living with and beyond cancer

'Screening for distress is recommended following a cancer diagnosis but the evidence base is unclear', said Alex Mitchell of the University of Leicester, Leicester, UK.

Distress/depression is severe in around 15% of cancerpatients, with 20% experiencing moderate symptoms, and 20% experiencing mild distress and depression. However, often the clinician has difficulty in recognising the symptoms of distress. Two large studies indicate that over 80% of late stage cancer patients suffer distress. Non-specialists who do not have a background in mental health issues have difficulty in ruling out distress.

Outcomes could be improved by screening of patients with follow-up enhanced psychosocial care and through increased psychosocial referral before and after distress screening.

Following screening and identification of distressed patients, only 36.5% of patients were willing to accept professional psychosocial help. Screening can influence uptake of care, but the effect is modest and barriers must be addressed for screening to be a success.These barriers include time, cost, and interest. Another important barrier is that even when the clinician identified distress, only 40% of them actually did anything in terms of offering the patients help. So psychological screening can work, but the barriers to access must be addressed.

Afaf Girgis of the University of New South Wales, Sydney, Australia discussed patients’ unmet care needs.

Patient-reported satisfaction levels are increasingly important foroffering high quality and specific patient-centred care. Systematic assessment and management of cancer patients’ unmet needs is an essential component of health care for people with cancer. Needs assessment tools (NAT) exist, and they have been developed and tested and should be used to find out if patients have specific needs. There are also NATs for the caregivers and in both cases these tools identify the unmet need in the cancer patient. Information about the prevalence and types of unmet needs being reported can inform service planning and redesign as well as patient-centred individual care. Importantly, there was no increase in consultation time if the NAT was used.

The most frequently reported needs include psychosocial, information, psychological, etc. It is important to remember that the clinicians tend to underestimate the psychological needs of the patient. There are no shortage of tools for needs assessment with over 35 tools available today.

PROMPT-Care assessments have been developed by this group to understand how needs change over time, and what are the predictors for this.The information gained is used to help self-management of the patient and also to implement timely clinical care.

Galina Velikova of St James’s University Hospital, Leeds, UK presented a proffered paper on a pilot study of REPORT-UK (Real-time Electronic Patient Outcome ReportTing of adverse events in UK clinical trials).

In clinical trials it is important that adverse events are reported; however, the standard system, the Common Toxicity Criteria and Adverse Events (CTCAE), relies heavily on the clinicians’ interpretation of adverse events. These reports often focus on safety rather that adverse effects.

The value of patient self-reports of adverse events (AEs) and Patient Reported Outcome Measures (PROMs) is recognised, but robust data collection methods are needed, particularly for clinical trials.

REPORT-UK was a proof-of-principle study to develop and evaluate an electronic (Internet/telephone) system for self-reporting AEs and PROMs during trials. A feasibility pilot study to assess compliance with the systems in a general oncology setting ran between August 2014 and October 2015.

In this study 249 cancer patients undergoing chemotherapy or radiotherapy/surgery were included. These individuals were followed for 12 weeks and were reminded to complete weekly AEs.

'We found that the consent rate was 48%', Dr Velikova said. Thirteen participants withdrew and six died whilst on study. The systems were perceived as easy-to-use. Time to complete was perceived by patients to be acceptable, and the Internet method of reporting was quicker compared to telephone. Patient and clinician-reporting of some AEs differed significantly.The study demonstrates a user-friendly electronic data collection system which provides information on patient compliance in a general oncology setting. The system could be implemented in practice in clinical trials alongside traditional approaches to improve data quality and safety.

## The epidemiology of obesity and hepatocellular carcinoma

Quentin Anstee of Newcastle University, Newcastle, UK presented on genetic factors in non-alcoholic fatty liver disease.

Hepatocellular carcinoma (HCC) incidence is increasing in part because of obesity. Non-alcoholic fatty liver disease (NAFLD) is often associated with HCC, potentially as an underlying aetiology. NAFLD ranges from steatosis, steatohepatitis (NASH), fibrosis, and ultimately cirrhosis and HCC.

Genome-wide association studies (GWAS) have led to a better understanding of the molecular factors contributing to the heterogeneity of this disease. The genetic modifiers PNPLA3 and TM6SF2 were found to be involved in disease pathogenesis. Anstee’s group have identified DNA methylation patterns that may also be involved in risk prediction of HCC. This may be a potential biomarker.

The aetiology of liver cancer is associated with increased fat uptake, alcohol uptake, and a sedentary lifestyle, explained Mathias Heikenwälder of the German Cancer Research Centre, Heidelberg, Germany. He says it affects around 90 million Americans and 30 million Europeans. Obesity has 'become pandemic,' not only in the US but in many European countries. Meanwhile, there is no effective pharmaceutical therapy for HCC.

The progression from NAFLD through to NASH and cirrhosis and onwards to HCC is important to understand. A 'two-hit hypothesis' has been proposed for NASH progression from NAFLD, lipid accumulation being the first step. The second hit,i.e., promotion of inflammation, DNA damage, hepatocyte cell death and fibrosis, is required for progression to NASH.

Dr Heikenwälder discussed a model for the NAFLD-NASH-cirrhosis-HCC progression in mice.In this mouse model NASH can be induced by a high fat diet in combination with methionine/choline-deficient diet (MCD). However, mice fed with MCD did not develop obesity, metabolic syndrome, or HCC, and the diet had to be discontinued after a few months because of weight loss. This model partially mimics what we see in NASH in humans. This approach allows one to study a chronic mouse model of NASH and eventual HCC without any genetic manipulation. These mice did eventually develop ‘real’ HCC and the model recapitulates what happens in the human metabolic syndrome, and this allowed the group to study gene amplification patterns in this disease. Further using this mouse model they demonstrated that after 12 months the mice did not have lipid deposits in the liver even though they were obese.HCC is the sixth most common cancer worldwide, said Dr Heikenwälder, and its high mortality rate is linked to this lack of effective therapy. Thus, reduction of lifestyle risk factors is key. An understanding of the disease progression will improve outcomes.

There may be no current effective therapeutic treatment for HCC, but there are promising advances in immunotherapy. Tim Meyer of the University College London, London, UK repeated that HCC is the sixth most common cancer worldwide and most patients will die of it.

He presented an overview of the treatment landscape of HCC. Sorafanib is the standard therapy but has little efficacy. Immunotherapy has a potential in liver cancer. It is known that patients with a high lymphatic infiltrate (CD4/8) have a better survival benefit. Peptide vaccinations increased survival slightly. Dendritic cell vaccines are of no real benefit. Adoptive cell therapies have not had any results in HCC. Checkpoint inhibitors are a potential direction of research.

Some promising results from a phase l/ll trial of nivolumab indicated that disease control was fairly good at nine months in HCC; however, it is too early for survival data. How effective these emerging treatments are remains to be seen. Controlling the underlying aetiology—from preventing obesity and lifestyle factors to understanding the mechanism of disease progression—appears to be the way forward with HCC.

Echoing the prevention theme in earlier talks, Gillian Rosenberg of Cancer Research UK (CRUK), London, UK hammered this message home with her proffered paper on the prevention of cancer in later life. In her opinion, obesity is the largest risk factor for cancer after tobacco. Obesity is linked to ten cancer types, she said, andoverweight children are more likely to become overweight adults.

Tobacco control programmes are known to be effective, and Rosenberg’s group at CRUK are trying to impose a similar programme for obesity. An obesity awareness study they conducted indicated that in general only 25% of individuals in the UK are aware of a link between being overweight and cancer. For childhood obesity, it is known that junk food marketing is associated with children wanting these foods. The aim is to prohibit these adverts until after the 9 pm watershed. 'Cancer Research UK aims to directly influence government policymaking in obesity, for example, encouraging the proposed sugar levy', Rosenberg said.

In conclusion this study addressed the poor knowledge of obesity and cancer in the general population. The mechanisms behind the causal relationship of obesity and cancer were identified. The childhood obesity studies demonstrated to policymakers the importance of taking action to limit advertising exposure in children. This linked back to the sessions on prevention, with the take-home message coming full circle—that prevention is at the population level

## Cancer communication

Along with developments in cancer research, another important topic of discussion at the conference was how cancer is talked about. Whether communicating to patients or the general public several speakers identified the problems, challenges, and opportunities that arise in communication about cancer.

This began on Sunday evening with a plenary lecture from Kat Arney, freelance science writer and science broadcaster, London. Kat discussed how media portrayal of the causes and treatments of cancer can influence public perception of cancer research. She described the need for the academic community to engage with the media and thereby help to shape the content reported in an accurate and engaging way.

Elena Semino from Lancaster University hosted a parallel session focussing on ‘How we talk about cancer’. The various speakers in this section reflected on their personal and professional experiences of the language used in cancer prevention; living with cancer; cancer treatment, and survivorship. Discussion also covered the language health professionals’ use and the part this plays in shaping peoples’ cancer experience and suggested a need for professionals to move toward a common language based on mutual understanding and meaningful partnership with the patient.

## Conclusion

During last year’s NCRI, our correspondents wondered how the social media angle would continue to develop. In 2014 and 2015, social media use at NCRI seemed geared towards providing a completely new area of open commentary and discussion. This year, the Twitter feed (**#NCRI2016**) and blogosphere continued in the same pattern as previous years. However, in her opening commentary, conference chair Prof Caroline Dive reminded social media users not to publicise unpublished work—clearly best practice for all!

Those who had downloaded the NCRI app were able to ask questions of the speakers over the Internet which were then collected at the end of sessions and presented to speakers. This streamlined the formerly clunky process of discussion especially for those who wanted to use the app. The anonymity offered by the app did appear to be more efficient than the method used in previous years which involved prospective questioners raising their hands, so that ushers could bring wireless microphones to them.

Precision oncology continues to be a meaningful discussion, and once again resistance and evolution were key topics. Prevention, especially at the population level, was a theme that connected many talks. The most prominent being the increased awareness of the relationship between obesity and cancer.

The take-home message for our correspondents this year was that the United Kingdom is increasingly aware of the need for prevention.

